# Human amniotic mesenchymal stem cells-conditioned medium protects mice from high-fat diet-induced obesity

**DOI:** 10.1186/s13287-021-02437-z

**Published:** 2021-06-26

**Authors:** Hui-Lan Tan, Xiao-Hui Guan, Min Hu, Jie Wu, Rong-Zhen Li, Ling-Fang Wang, Hou-Da Huang, Zhen-Ping Yu, Xiao-Yu Wang, Yun-Fei Xiao, Ke-Yu Deng, Hong-Bo Xin

**Affiliations:** 1 The National Engineering Research Center for Bioengineering Drugs and the Technologies, Institute of Translational Medicine, Nanchang University, No. 1299 Xuefu Road, Honggutan District, Nanchang, 330031 China; 2 School of Pharmacy, Nanchang University, Nanchang, China; 3 School of Life and Science, Nanchang University, Nanchang, China

**Keywords:** Obesity, hAMSCs-CM, Energy expenditure, Lipid metabolism, Glucose metabolism, Inflammation

## Abstract

**Background:**

Obesity is a metabolic disorder syndrome characterized by excessive fat accumulation that is related to many diseases. Human amniotic mesenchymal stem cells (hAMSCs) have a great potential for cell-based therapy due to their characteristics such as pluripotency, low immunogenicity, no tumorigenicity, potent paracrine effects, and no ethical concern. Recently, we observed that both hAMSCs and their conditioned medium (hAMSCs-CM) efficiently repaired skin injury, inhibited hepatocellular carcinoma, and alleviated high-fat diet (HFD)-induced diabetes. However, the effects and the underlying mechanisms of hAMSCs-CM on high-fat diet (HFD)-induced obesity were not explored.

**Methods:**

The characteristics of hAMSCs were confirmed by flow cytometry, RT-PCR, and immunofluorescence. Obese mice were induced by administrating HFD for 15 weeks and simultaneously, the mice were intraperitoneally injected with hAMSCs-CM weekly to evaluate the effects of hAMSCs-CM on HFD-induced obesity. GTT and ITT assays were used to assess the effects of hAMSCs-CM on HFD-induced glucose tolerance and insulin resistance. The lipid accumulation and adipocytes hypertrophy in mouse adipose tissues were determined by histological staining, in which the alterations of blood lipid, liver, and kidney function were also examined. The role of hAMSCs-CM in energy homeostasis was monitored by examining the oxygen consumption (VO_2_), carbon dioxide production (VCO_2_), and food and water intake in mice. Furthermore, the expressions of the genes related to glucose metabolism, fatty acid β oxidation, thermogenesis, adipogenesis, and inflammation were determined by western blot analysis, RT-PCR, and immunofluorescence staining. The roles of hAMSCs-CM in adipogenesis and M1/M2 macrophage polarization were investigated with 3T3-L1 preadipocytes or RAW264.7 cells in vitro.

**Results:**

hAMSCs-CM significantly restrained HFD-induced obesity in mice by inhibiting adipogenesis and lipogenesis, promoting energy expenditure, and reducing inflammation. The underlying mechanisms of the anti-obesity of hAMSCs-CM might be involved in inhibiting PPARγ and C/EBPα-mediated lipid synthesis and adipogenesis, promoting GLUT4-mediated glucose metabolism, elevating UCP1/PPARα/PGC1α-regulated energy expenditure, and enhancing STAT3-ARG1-mediated M2-type macrophage polarization.

**Conclusion:**

Our studies demonstrated that hAMSCs significantly alleviated HFD-induced obesity through their paracrine effects. Obviously, our results open up an attractive therapeutic modality for the prevention and treatment of obesity and other metabolic disorders clinically.

**Graphic Abstract:**

The cytokines, exosomes, or micro-vesicles secreted from hAMSCs significantly inhibited HFD-induced obesity in mice by inhibiting lipid production and adipogenesis, promoting energy consumption, and reducing inflammation. 
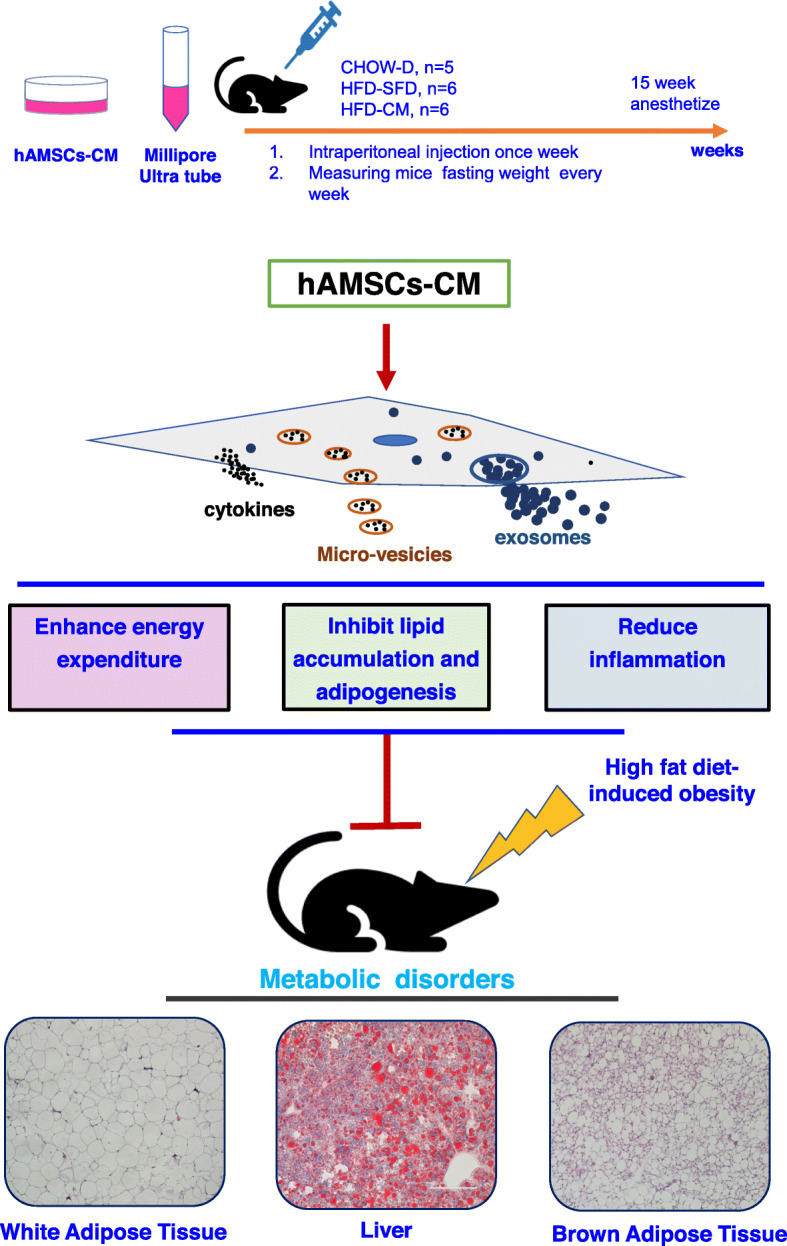

**Supplementary Information:**

The online version contains supplementary material available at 10.1186/s13287-021-02437-z.

## Background

Obesity is a metabolic disorder syndrome characterized by excessive fat accumulation in the body due to excessive food intake or the alteration of lifestyle and it is a risky factor for many diseases such as diabetes and cardiovascular diseases [1, 2]. In general, the occurrence of obesity is closely related with energy homeostasis, lipogenesis, and inflammation [3].

Metabolic disorder is a predisposition factor of obesity. Peroxisome proliferator-activated receptors α (PPARα) and PPAR-coactivator 1 α (PGC1α) play an important role in energy homeostasis such as fatty acid β oxidation and gluconeogenesis [4, 5]. It has been reported that fasted PPARα^−/−^ mice were not able to sufficiently catabolize lipids in the liver and finally resulted in steatosis [6]. Glucose is a main energy source for mitochondrial metabolism especially in adipose and muscle tissues. In dysfunctional adipose tissues, the expressions of glucose transporters (GLUTs) were remarkably decreased, resulting in insulin resistance-induced hyperglycemia [7]. Hence, correction of energy homeostasis disorder is an effective way to prevent obesity.

Adipose tissues include white adipose tissues (WAT) and brown adipose tissues (BAT) which possess different functions [8]. WAT primarily serves as an important organ for energy storage, but also is able to secrete numerous hormones in response to nutritional signals [9]. In contrast, BAT, as a thermogenic organ, is responsible for converting calorie to heat in cold circumstances [10], in which the processes are mainly mediated by activating mitochondrial uncoupling protein 1 (UCP1) which is a mitochondrial protein uncoupling cellular respiration and ATP synthesis to dissipate energy as heat [11]. Therefore, BAT inhibits obesity or obesity-related metabolic diseases by promoting glucose and lipid consumption [12]. PPARγ, carbohydrate response element-binding protein (C/EBP), and sterol response element-binding protein 1c (SREBP1c) are vital transcriptional regulators in adipogenesis [4, 13, 14]. WAT, as a secretory organ, plays a crucial role in the adipose tissue inflammation by secreting numerous adipokines to recruit macrophages into the tissues [15, 16]. Studies also revealed that eliminating the adipose tissues inflammation could alleviate obesity [17]. Therefore, several strategies such as enhancing energy expenditure, inhibiting adipogenesis, and reducing adipose tissues inflammation can be used for preventing or treating obesity.

Studies showed that mesenchymal stem cells (MSCs)-derived conditioned medium (CM) and exosomes contained abundant cytokines, growth factors, extracellular matrix proteins, chemokines, and nuclear acids [18]. It has been reported that MSCs-CM effectively treated many diseases such as pulmonary disease, hair regrowth, diabetes, liver fibrosis, hepatoma carcinoma, osteoarthritis by inhibiting inflammation, and promoting immuno-regulation [19–22].

Human amniotic mesenchymal stem cells (hAMSCs) are considered as a promising source of stem cells for cell-based therapy due to their characteristics such as pluripotency, low immunogenicity, no tumorigenicity, potent paracrine effects, and no ethical concern [23]. Previously, we observed that both hAMSCs and their CM efficiently repaired skin injury [24], inhibited hepatocellular carcinoma [25], and alleviated HFD-induced hyperglycemia in mice (unpublished data). It has been reported that adipose MSC-derived exosomes ameliorated HFD-induced obesity in mice through inhibiting inflammation, improving insulin sensitivity, and decreasing hepatic steatosis [26, 27]. However, the effect of hAMSCs-CM on HFD-induced obesity in mice was not explored. Based on the advantages of hAMSCs in the cell-based therapy and the complexity of obesity, we hypothesized that hAMSCs-CM might alleviate HFD-induced obesity through affecting energy expenditure, inhibiting adipogenesis, and reducing adipose tissue inflammation. Obviously, our results would provide a new insight in elucidating the mechanism of the anti-obesity of hAMSCs-CM and a new strategy for the prevention and treatment of metabolic disorder-related diseases such as obesity clinically.

## Methods

### Isolation, culture, and characterization of hAMSCs

Human amniotic membrane tissue of the placenta was obtained from healthy pregnant women (38–41 weeks) after they gave their births or when they were undergoing cesarean delivery in the First Affiliated Hospital of Nanchang University. Informed consent was obtained from women who voluntarily donated their placentas. The obtained tissue samples were only used for scientific research, not for commercial and reproductive ethics-related research and development. The research procedure was approved by the ethics committee of the First Affiliated Hospital of Nanchang University.

hAMSCs were isolated and cultured as described [24, 28]. Briefly, amnions isolated from human placenta were cut into small pieces and washed with D-Hanks buffer containing 100 U/mL penicillin and 100 μg/mL streptomycin and then digested with 0.25% trypsin-EDTA and type IV collagenase (1 mg/mL; Worthington, Lakewood, NJ) respectively. Then, the cells were cultured in athe complete medium. The supernatants were discarded after 5 h incubation. The adherent cells were continued to be maintained in a complete medium at 37 °C with 5% CO_2_.

The hAMSCs were harvested to identify the immunophenotype using PE-conjugated anti-CD45, anti-CD90, anti-CD105, anti-CD29, and anti-CD40 antibodies and FITC-conjugated anti-CD73, anti-CD34, anti-ABC, anti-CD80, and anti-HLA-DR antibodies (all antibodies purchased from BD Biosciences) by flow cytometry analysis. The characteristics of hAMSCs were also confirmed by RT-PCR and immunofluorescence.

### Preparation of hAMSCs-CM

The complete medium of hAMSCs was replaced with the serum-free DMEM (SFD) when the cell density reached to 80–90% confluence. The supernatants were collected after 48 h and centrifuged at 1500 rpm for 5 min to remove all cellular debris, and the cell numbers were counted. The supernatant which was referred as hAMSCs-CM were further concentrated with a Millipore centrifuge tube (Millipore, UFC900324) and stored at − 80 °C for future experiments.

### Experimental animals

Six- to 8-week-old male C57BL/6 mice (Hunan SJA Laboratory Animal Co, Ltd.) were fed with a normal chow diet (CHOW-D, Jiangsu Xietong Pharmaceutical Bio-engineering Co, Ltd, C00105) or high-fat diet (HFD, D12492, 60% kcal from fat, Research Diet, USA) for 15 weeks, in which the mice were housed in a temperature (20–24 °C) and humidity-controlled (45–55%) individual ventilated cages with a 12 h/12 h light/dark cycle. Mice were randomly assigned into 3 groups, hAMSCs-CM-treated group with HFD (n = 6), SFD-treated group with HFD (n = 6), CHOW-D group (n = 5), and were intraperitoneally injected with hAMSCs-CM suspended in 0.4 mL DMEM (for hAMSCs-CM group) or 0.4 mL SFD alone (for SFD group) for 15 weeks. Fasting body weight was monitored weekly. The final administrated concentration of hAMSCs-CM for each mouse was defined as a concentration which was equivalent to the collected conditional medium of 1.5 × 10^6^ cells cultured in serum-free medium for 48 h.

### Energy expenditure measurement

Mice were accustomed into the metabolic chambers for more than 24 h before performing the measurement of metabolic parameters and then the oxygen consumption (VO_2_), carbon dioxide production (VCO_2_), and food and water intake were monitored for 48 h in animal monitoring system TSE PhenoMaster (Germany, TSE Systems) following the instruction of the manufacturer.

### Glucose tolerance test (GTT) and insulin tolerance test (ITT)

GTT and ITT were performed in mice by injection of glucose (2 g/kg) or insulin (0.5 U/kg), intraperitoneal injection (I.P.) after the mice were fasted for 16 h or 4 h, respectively, and the tail capillary blood glucose concentrations were measured with a glucometer (Sinocare, GA-3) at indicated times.

### Biochemical analysis

Serum alanine transaminase (ALT), aspartate aminotransferase (AST), total cholesterol (TC), triglyceride (TG), high-density lipoprotein cholesterol (HDL-C), low-density lipoprotein cholesterol (LDL-C), total protein (TP), creatinine (CREA), and blood urine nitrogen (BUN) were detected using automatic biochemical analyzer (Beckman Coulter AU2700) according to Beckman AU clinical biochemical reagent.

### Immunofluorescence staining

WAT and BAT were fixed with 4% PFA (Solarbio, Beijing, P1110) and embedded in paraffin. The 4-μm sections were prepared, followed by deparaffinization, rehydration, and quenching endogenous peroxidases with 0.3% H_2_O_2_ (in PBS) for 10 min. Then the tissue sections were incubated with antigen retrieval buffer (Solarbio, C1032) at 95 °C for 20 min and were permeabilized by 0.1% Triton X-100 and incubated with anti-F4/80 antibody (Abcam, 1:100, ab6640) and UCP1 antibody (Abcam, 1:500, ab23841) overnight at 4 °C followed by incubating with a secondary antibody goat anti-rat IgG H&L (Alexa Fluor® 488) and goat anti-rabbit IgG H&L (Alexa Fluor® 647) for 60 min. Nuclei were stained with DAPI (Solarbio, S2110). hAMSCs were fixed with 4% PFA for 30 min at RT and blocked with 10% goat serum for 60 min. Primary antibodies against SSEA4 (Abcam, 1:100, ab16287) or Vimentin (Proteintech, 1:200, 10366-1-AP) were then incubated overnight at 4 °C. After that, cells were incubated with secondary antibody goat anti-mouse or anti rabbit (Alexa Fluor® 488) for 60 min.

### Hematoxylin-eosin (HE) staining

Adipose tissues were isolated from the mice and fixed with 4% PFA overnight. The tissues embedded in paraffin were sectioned into 4 μm thickness, followed by deparaffinization, rehydration, and finally stained with hematoxylin and eosin following the manufacturer’s introductions (Solarbio, Beijing, G1121).

### Periodic acid-Schiff (PAS) staining

Liver sections were rehydrated according to the HE staining procedures and washed with PBS and incubated with Schiff reagent (Solarbio, Beijing, G1280) for 15 min. After rinsing with distilled water for 2 min, the sections were dyed with hematoxylin, and glycogen deposition in the liver was showed in purple.

### Cell culture

Pre-adipocytes cell line 3T3-L1 and RAW264.7 cells were purchased from Stem Cell Bank, Chinese Academy of Sciences. 3T3-L1 cells were cultured with DMEM (Gibco, C11995500BT) containing 10% new bovine serum. RAW264.7 cells were cultured with RPMI-1640 (Gibco, C1187500BT) containing 10% fetal bovine serum (BI, 04-004-1A). hAMSCs were cultured in MEM-α (Gibco, A10490-01) containing 10% fetal bovine serum (Gibco, 10099-141), 2% Chang C (IrvineScientific, C108), 18% Chang B (IrvineScientific, C100), 0.1%FGF-basic (20 ng/mL, Peprotech, 100-1B), 1% L-GLU (Gibco, 35050061), and 0.1% β-Mercaptoethanol (Solarbio, Beijing, 21985023). All cells were cultured with 100 U/mL penicillin and 100 mg/mL streptomycin and maintained at 37 °C in a humidified incubator under 5% CO_2_. One hundred ng/mL LPS (Sigma-Aldrich, L4130), 2.5 ng/mL IFN-γ (Novoprotein, C746), and 10 μg/mL IL-4 (Novoprotein, CK74) were used in the experiments of RAW264.7 cells.

### Differentiation of 3T3-L1 pre-adipocytes

3T3-L1 preadipocytes were cultured and maintained in DMEM containing 10% fetal calf serum. When the cells reached to 100% confluence, the 3T3-L1 preadipocytes were cultured for another 48 h. Then the differentiation of 3T3-L1 preadipocytes were induced with an induction medium containing 0.5 mM IBMX (Sigma-Aldrich, 15879), 1 μM dexamethasone (Solarbio, Beijing, D8040), and 10 μg/mL insulin (Jiangsu Wanbang Biopharmaceutical Co, Ltd.) for 2 days. Subsequently, the cells were maintained in an adipocyte culture medium supplemented with 10 μg/mL insulin for another 2 days and replaced with fresh 10% FBS complete medium every 2 days. After 14 days induction, obvious cellular lipid droplets were observed.

### Oil Red O staining

Liver tissues were fixed in 4% PFA and then were gradually incubated in gradient sucrose solutions (30%, 50%, and 70% sucrose) for more than 12 h until liver tissues sank into the bottom, and then the tissues were embedded with OCT compound (Tissue Tek, 4583) and 8-μm frozen sections were used for Oil Red O staining (Sigma-Aldrich, 1320-06-5). The differentiated mature adipocytes were washed with PBS and fixed with 4% PFA overnight and the cells were incubated with working Oil Red O solution (in 60% isopropanol) for 10 min. After washing twice with PBS, the cells were visualized under an inverted microscope (Olympus, Tokyo, Japan).

### RNA isolation, cDNA synthesis, and quantitative real-time PCR

Total RNAs from adipose tissues or cells were isolated using TRIzol reagent (Life technology, 15596018) according to the manufacturer’s instructions. Single-stranded cDNA were synthesized with a reverse transcription kit (Thermo Fisher Scientific, K1622). mRNA levels were measured by qRT-PCR with SYBR-RT-PCR master mix (Roche, USA) with ABI ViiA7 PCR machine (Applied Biosystems, USA). GAPDH was used as a loading control. The analyses of the related gene expressions were performed with the relative quantification comparative CT method. The primer sequences for qRT-PCR were shown in Supplementary Tab. 1.

### Western blot

Adipose tissues were lysed with RIPA buffer (Solarbio, Beijing, R0010), and protein concentrations were determined by a BCA Protein Assay Kit (Thermo Fisher Scientific, 23227). Each sample containing 40 μg protein was loaded in SDS PAGE gel for western blot analyses and the primary antibodies were used as follows: SREBP1 (Santa Cruz, rabbit,1:250, sc-8984), PPARγ (Santa Cruz, mouse, 1:250, sc-7273), FASN (Abcam, ab128870, 1:5000), C/EBPα (Santa Cruz, rabbit, AF6333, 1:500), GLUT4 (1:1000, rabbit, CST, 2213), P-AKT (1:1000, rabbit, CST, #9272), AKT (1:1000, rabbit, CST, #4060), PPARα (Santa Cruz, mouse, sc-398394, 1:1000), PGC1α (Santa Cruz, rabbit, sc-13067, 1:1000), ARG-1 (1:1000, rabbit, CST, 93668 s), STAT3 (1:1000, rabbit, CST, 4904 s), P-STAT3 (1:1000, rabbit, CST, 9145), β-actin (1:5000, mouse, ABclonal, AC004), and GAPDH (1:10000, mouse, KANGCHEN, KC-5G4). The secondary antibodies were used as follows: HRP-conjugated goat anti-rabbit (1:5000, Thermo Fisher Scientific, 31460) and goat anti-mouse (1:5000, Thermo Fisher Scientific, 31430) antibodies. β-actin or GAPDH was used as a reference control. Signals were detected using the ECL Plus detection system (Thermo Fisher Scientific) and the images were quantified with ImageJ software.

### Statistical analysis

The results were expressed as means ± SD (standard deviation). The statistical analysis was carried out using the Graphpad Primer 5 software. Differences between groups were assessed using Student’s t tests (two samples) or one-way ANOVA (three or more samples). For all analyses, the statistical significance level was set at *p/^**#**^*p <* 0.05; **p/^**##**^*p <* 0.01, ***p/^**###**^*p <* 0.001.

## Results

### Characteristics of hAMSCs

**Fig. 1:**
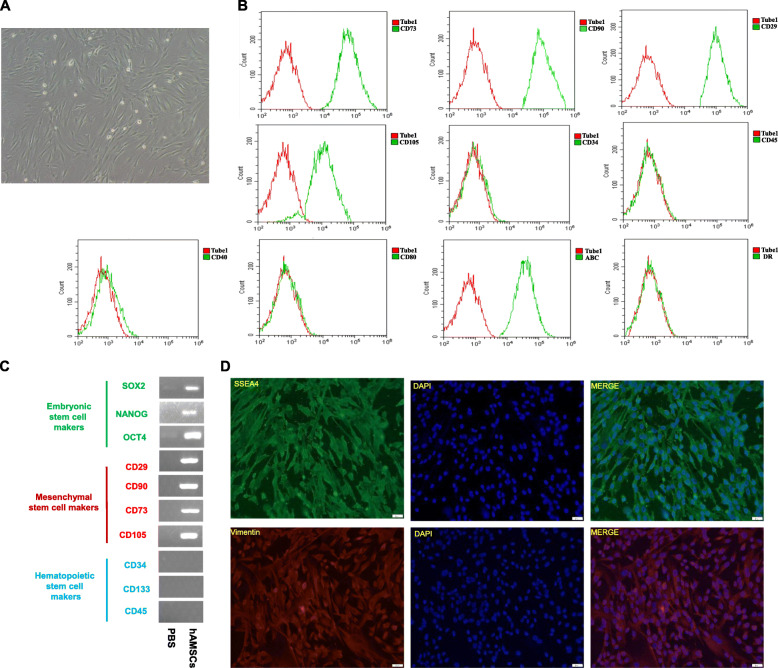
Characteristics of human amniotic mesenchymal stem cells (hAMSCs). **a** hAMSCs showed a spindle-like and fibroblastic-shaped morphology (scale bar = 100 μm). **b** The expressions of the featured makers in hAMSCs were detected by flow cytometric analysis for mesenchymal stem cells (CD73, CD90, CD29, CD105), hematopoietic stem cells (CD34, CD45) and human leukocyte antigen class I (HLA-I ABC), co-activator cytokine (CD40, CD80), and human leukocyte antigen class II (HLA-II DR). The expression of each antigen was presented with the corresponding isotype control (tube 1 in red). **c** The expressions of various markers were confirmed by PCR: embryonic stem cell surface markers of SOX2, Nanog, and OCT4; mesenchymal stem cell markers of CD29, CD90, CD73, and CD105 and hematopoietic stem cell markers of CD34, CD133, and CD45. **d** SSEA4 (embryonic stem cells surface marker) and Vimentin (mesenchymal stem cells marker) were detected by immunofluorescent staining in hAMSCs (scale bars, 20 μm)

hAMSCs were isolated from the human amniotic membrane as previously described [24] and the hAMSCs have a typical bipolar spindle-like and fibroblastic-shaped morphology (Fig. 1A). Flow cytometry showed that hAMSCs expressed mesenchymal stem cells (MSCs) surface markers including CD73, CD90, CD29, and CD105, as well as human leukocyte antigen class I (HLA-I ABC). However, hAMSCs did not express CD40, CD80, HLA-II DR, and hematopoietic stem cell (HSC) markers CD34 and CD45 (Fig. 1B). RT-PCR assay further confirmed that hAMSCs did express the embryonic stem cell (ESC) markers (SOX2, Nanog, OCT4) and MSC markers (CD29, CD90, CD73, CD105), but not express HSC markers (CD34 CD133, CD45) (Fig. 1C). In addition, the expressions of ESCs surface marker SSEA4 and MSCs marker Vimentin were also confirmed by immunofluorescent staining (Fig. 1D). All these results indicated that hAMSCs possess the potentials of the multi-lineage differentiation with low immunogenicity.

### hAMSCs-CM significantly alleviated high-fat diet-induced obesity

**Fig. 2:**
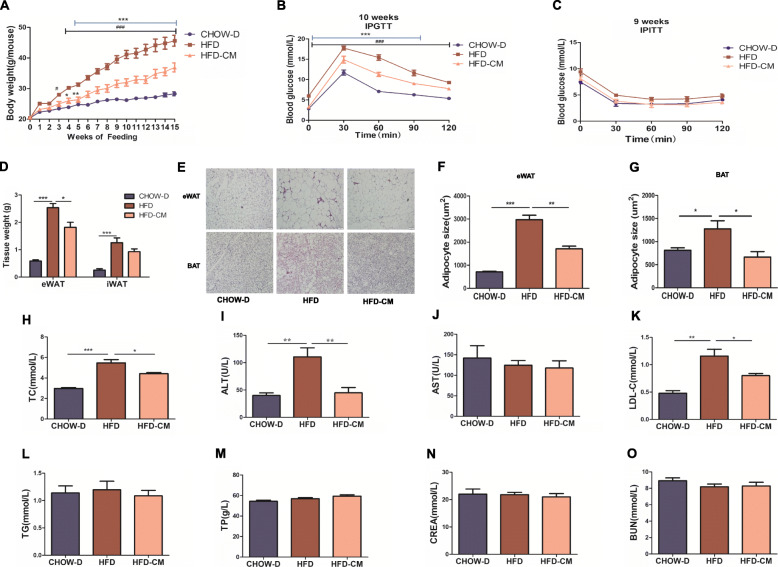
Effects of hAMSCs-CM on HFD-induced obesity in mice. **a** Mice were fed with HFD or CHOW for 15 weeks and were intraperitoneally injected with hAMSCs-CM suspended in 0.4 mL serum-free DMEM (SFD) (for the hAMSCs-CM group) or 0.4 mL SFD alone for the HFD group every week and their body weight were monitored every week. **b**, **c** Intraperitoneal insulin tolerance test (IPITT, right) and intraperitoneal glucose tolerance test (IPGTT, left) were performed at ninth or tenth week, respectively. Blood glucose concentration was determined by chemstrips and a glucometer through tail vein. **d** Weights of eWAT and iWAT were quantitatively analyzed. **e** Images of WAT and BAT were analyzed by HE staining (scale bars, 50 μm). **f**, **g** Sizes of adipocytes were quantitatively analyzed in WAT (**f**) and BAT (**g**). Serum total cholesterol (TC, **h**), alanine transaminase (ALT, **i**), aspartate aminotransferase (AST, **j**), low-density lipoprotein cholesterol (LDL-C, **k**), and triglycerides (TG, **l**), total protein (TP, **m**), creatinine (CREA, **n**) and blood urea nitrogen (**o**, BUN) were measured using automatic biochemical analyzer (Beckman Coulter AU2700). Data represent as means ± SD. #*p<* 0.05, ##*p<* 0.01, ###*p*< 0.001 and *p< 0.05, **p< 0.01, ***p< 0.001 represents CHOW-D vs HFD and HFD vs HFD-CM, respectively

Our recent work showed that both hAMSCs and their conditioned medium (hAMSCs-CM) were able to attenuate high-fat diet (HFD)-induced hyperglycemia, indicating that their conditioned medium might play a key role in HFD-induced hyperglycemia (unpublished data). Here, we investigated the effects of hAMSCs-CM on HFD-induced obesity in mice. hAMSCs-CM or SFD was intraperitoneally injected weekly into the mice fed with HFD and the body weights were monitored every week. As showed in Fig. 2A, HFD-induced gains of body weights were significantly reduced in the mice treated with hAMSCs-CM compared with mice treated with SFD. The results of GTT and the ITT assays showed that HFD-influenced glucose tolerance and insulin sensitivity were significantly improved in the mice treated with hAMSCs-CM compared with the mice treated with SFD (Fig. 2B, C), further confirming that HFD-induced obesity was accompanied with hyperglycemia and the anti-obesity of hAMSCs-CM might be related with improvement of HFD-induced glucose tolerance and insulin resistance. In addition, hAMSCs-CM remarkably reduced HFD-induced gains of adipose tissues such as epididymal and inguinal white adipose tissues (eWAT and iWAT, Fig. 2D) and protected mice from HFD-induced enlargements of adipocytes in WAT and BAT compared with mice treated with SFD (Fig. 2E–G). Furthermore, the HFD-induced increases of serum TC (Fig. 2H), ALT (Fig. 2I), and LDL-C (Fig. 2K) were markedly reduced in the hAMSCs-CM group compared with the SFD group, suggesting that hAMSCs-CM might improve HFD-induced the dysfunctions of the liver. However, there were no alterations in contents of serum AST (Fig. 2J), TG (Fig. 2L), TP (Fig. 2M), CREA (Fig. 2N), and BUN (Fig. 2O) in various groups after HFD stimulation, suggesting that HFD might not significantly affect kidney functions. All these results indicated that hAMSCs-CM significantly alleviated HFD-induced obesity in mice.

### hAMSCs-CM ameliorated HFD-induced obesity by promoting energy expenditure and improving liver functions

**Fig. 3:**
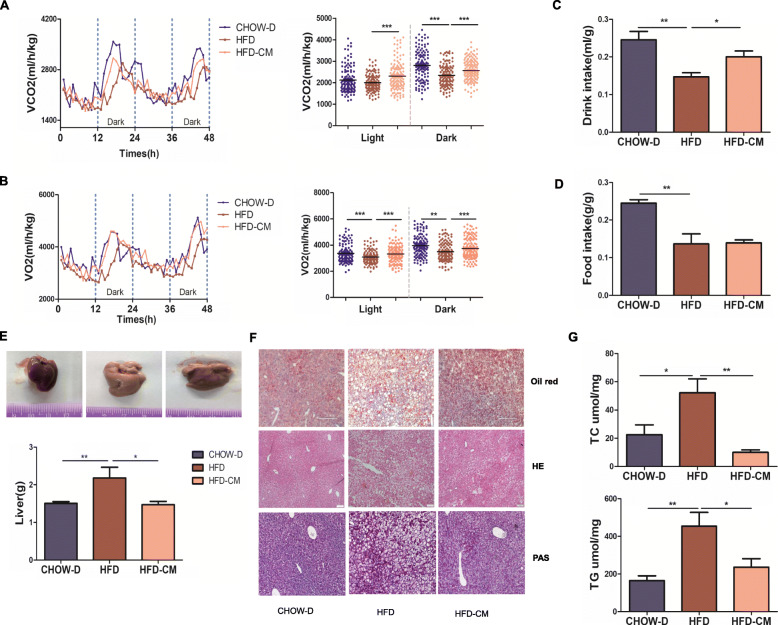
Effects of hAMSCs-CM on HFD-induced alterations of energy expenditure in mice and lipid accumulation of liver in mice. **a**–**d** Mice were placed into the metabolic chambers for more than 24 h before measuring metabolic parameters. The energy expenditure was evaluated by measuring the carbon dioxide release (VCO_2_, **a**) oxygen consumption (VO_2_, **b**), and water (**c**) and food (**d**) intake in mice fed with HFD and normal diet with or without hAMSCs-CM treatment. **e** The images of livers were taken in the control group (left), SFD group (medium), and hAMSCs-CM group (right), and the weights of liver tissues were quantitatively analyzed (bottom). **f** The effects of hAMSCs-CM on HFD-induced lipid accumulation, alterations of morphology, and the glucagon production were determined by Oil Red staining, HE staining, and periodic acid-Schiff (PAS) staining in liver tissues (scale bars, 200 μm). **g** Effects of hAMSCs-CM on HFD-induced the increases of TC and TG contents were determined in liver tissues. Data represent as means ± SD. N = 5~6, *p< 0.05; **p< 0.01, ***p< 0.001

The main feature of obesity is the metabolic disorder which is closely related with the functional alterations of the liver. In order to evaluate the roles of hAMSCs-CM in energy homeostasis, we examined the oxygen consumption (VO_2_), carbon dioxide production (VCO_2_), and food and water intake in mice for 48 h using TSE PhenoMaster (Germany, TSE Systems). The results showed that HFD-induced reductions in VO_2_, VCO_2_, and water intake were significantly reversed by hAMSCs-CM in mice fed with HFD compared with mice treated with SFD (Fig. 3A-D), indicating that hAMSC-CM effectively alleviated HFD-induced adiposity in mice by increasing energy expenditure.

Next, we examined the alterations of liver morphology and functions. The results showed that hAMSCs-CM markedly reduced HFD-induced gain of liver weights (Fig. 3E) and significantly improved HFD-induced histological alterations of the liver by decreasing lipid droplet deposition and increasing glycogen accumulation (Fig. 3F) which were revealed by HE staining, Oil Red staining, and PAS staining, respectively. Furthermore, the HFD-induced increases of the TC and TG in liver tissues were markedly reduced in the hAMSCs-CM group compared with the SFD group (Fig. 3G). These results suggested that the alterations of liver functions contributed to the HFD-induced obesity in mice.

### hAMSCs-CM improved HFD-induced metabolic dysfunctions by enhancing energy expenditure and elevating thermogenesis in mice

**Fig. 4:**
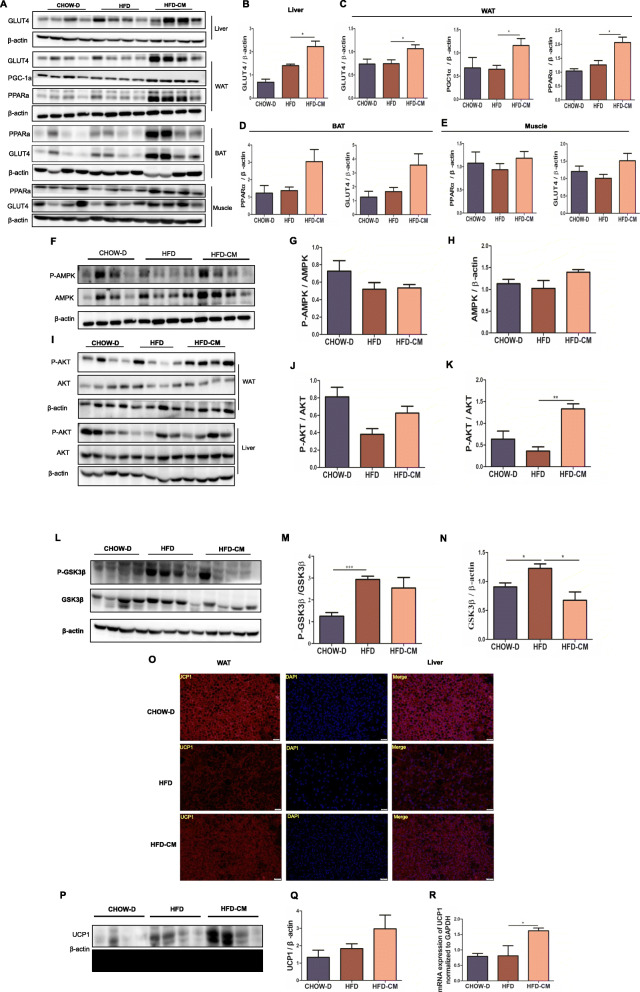
Effects of hAMSCs-CM on the expressions of the proteins related with lipid and glucose metabolism, and thermogenesis in multiple tissues of obese mice. **a** Expressions of GLUT4 in liver, PPARα and GLUT4 in WAT&BAT&muscle, and PGC-1α in WAT were examined by western blot. **b**–**e** The protein expressions of GLUT4 in the liver (**b**) & WAT (**c**) & BAT (**d**) & muscle (e), PPARα in WAT (**c**) & BAT (**d**) & muscle (**e**), and PGC-1α in WAT (**c**) were quantitatively analyzed. **f**–**h** the protein expressions of P-AMPK and AMPK in WAT were determined by western blot (**f**) and quantitative analyses (**g**, **h**). **i**–**k** The protein expressions of P-AKT and AKT in WAT and liver were determined by western blot (**i**) and quantitative analyses (**j**, **k**). **l**–**n** the protein expressions of P-GSK3β and GSK3β in the liver were determined by western blot (**l**) and quantitative analyses (**m**, **n**). **o** UCP1 expression was determined by immunofluorescent staining in BAT (scale bars, 20 μm). **p**, **q** The expression of UCP1 protein was determined by western blot (**p**) and quantitative analysis (**q**) in BAT. **r** UCP1 mRNA expression was also examined by RT-PCR in BAT. β-actin was served as the loading control. Data represent as means ± SD. *p< 0.05; **p< 0.01, ***p< 0.001

To investigate the roles of hAMSCs-CM in HFD-induced metabolic disorder, the glucose and lipid metabolism and thermogenesis were examined in obese mice. The results showed that the expressions of the glucose transporter GLUT4 in the liver, WAT, BAT, and muscle tissues; PPARα which is related to lipid metabolism in WAT, BAT, and muscle tissues; and PGC1α which is a cold-inducible transcription coactivator of adaptive thermogenesis in WAT (Fig. 4A–E) were markedly increased in HFD-fed mice treated with hAMSCs-CM compared with the mice treated with SFD (Fig. 4A–E). These results suggested that hAMSCs-CM reduced the transfer of glucose to lipid generation by promoting glucose utilization and glycogen synthesis. AMP-activated protein kinase (AMPK) is a crucial cellular energy sensor, which takes part in energy balance [29]. Our results also displayed that hAMSCs-CM had a trend of elevating AMPK protein expression in WAT (Fig. 4F–H), suggesting that hAMSCs-CM might reduce the HFD-induced lipid generation by activating AMPK signaling pathway. Akt phosphorylation was a classic insulin activation regulator, involved in glucose metabolism [30]. Furthermore, hAMSCs-CM significantly ameliorated HFD-induced reduction of phosphorylated AKT in WAT (Fig. 4I, J) and liver (Fig. 4I, K). Glycogen synthase kinase 3β (GSK3β) as a AKT downstream regulator plays a role in glycogen metabolism [31]. The results showed that hAMSCs-CM attenuated HFD-induced upregulation of phosphorylated GSK3β in the liver (Fig. 4L–N). These results further demonstrated that hAMSCs-CM promoted glucose metabolism or glycogen synthesis by activating AKT signaling pathway.

Reduced thermogenesis is a characteristic feature of obesity in mice and humans. UCP-1 is an important thermogenesis protein regulating energy metabolism in BAT. The results showed that UCP-1 was upregulated by IF staining assay (Fig. 4O). Similar results were also observed in protein and mRNA levels (Fig. 4P–R) in BAT. Taken together, these results indicated that hAMSCs-CM significantly improved HFD-induced metabolic dysfunction by promoting energy expenditure and thermogenesis in obese mice.

### hAMSCs-CM inhibited adipogenesis through suppressing the differentiation of the pre-adipocytes

**Fig. 5:**
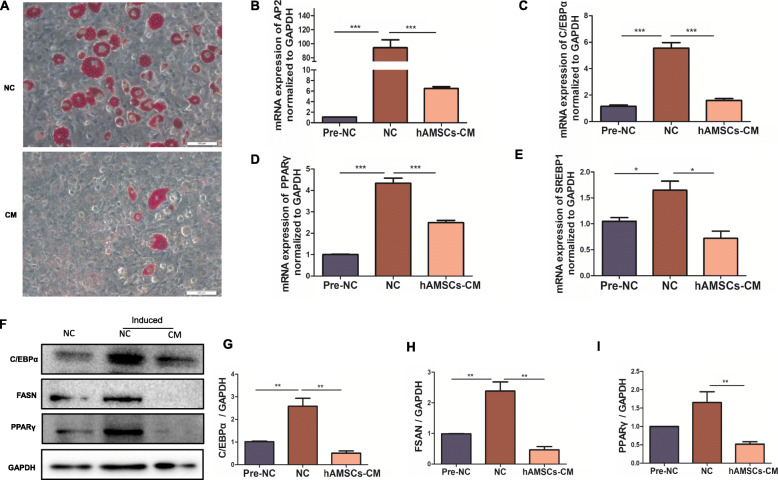
Effects of hAMSCs-CM on differentiation of pre-adipocytes 3T3-L1. **a** the lipid deposition was detected by Oil-O Red staining in pre-adipocytes 3T3-L1 after 14 days differentiation induction (scale bars, 100 μm). **b**–**e** the mRNA expressions of AP2, C/EBPα, PPARγ, and SREBP1 were examined by RT-qPCR. **f** The protein levels of C/EBPα, FASN, and PPARγ in 3T3-L1 cells were determined by western blot (GAPDH served as the loading control). **g-i** The protein levels of C/EBPα, FASN, and PPARγ were quantitatively analyzed. Each experiment was repeated three times. Data represent as means ± SD. *p< 0.05; **p< 0.01, ***p< 0.001

The occurrence of obesity is often accompanied with preadipocytes differentiation and adipogenesis. The differentiation from preadipocytes to mature adipocytes is a critical process for obesity development. Therefore, we examined the effects of hAMSCs-CM on the differentiation of 3T3-L1 preadipocytes into adipocytes in vitro. The results showed that hAMSCs-CM significantly inhibited preadipocytes differentiation and lipid accumulation after a 14-day period induction in 3T3-L1 cells (Fig. 5A). In addition, hAMSCs-CM also remarkably suppressed the mRNA expressions of the genes related to adipogenesis such as AP2, C/EBPα, PPARγ, and SREBP1 after differential induction (Fig. 5B–E). Furthermore, the protein expressions of C/EBPα, FASN, and PPARγ were also remarkably reduced in the hAMSCs-CM group compared with the SFD group (Fig. 5F–I). These results demonstrated that hAMSCs-CM was able to inhibit the preadipocytes differentiation in vitro.

### hAMSCs-CM suppressed WAT inflammation by activating STAT3/ARG-1 pathway or altering the macrophage polarization in the obese mice

**Fig. 6:**
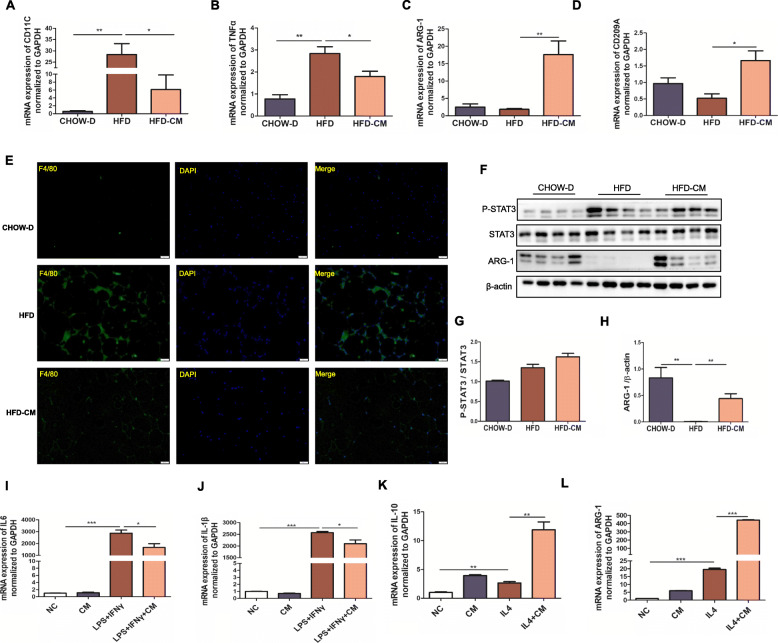
Effects of hAMSCs-CM on HFD-induced inflammation in WAT of the obese mice. **a**–**d** The mRNA expressions of the pro-inflammatory cytokines CD11C and TNFα (**a**, **b**) and the anti-inflammatory cytokines ARG-1 and IL10 (**c**, **d**) were detected by RT-PCR in WAT. **e** F4/80 expressions were determined by IF staining in WAT (scale bars, 20 μm). **f** The protein levels of P-STAT3, STAT3, and ARG-1 were detected by western blot. **g**, **h** The ratio of P-STAT3/STAT3 and the quantitative Arg-1 were analyzed in WAT (β-actin served as the loading control). **i**–**l** LPS+IFNγ or IL4-mediated the mRNA expressions of IL6, IL-1β, IL10 and ARG-1 were examined by RT-PCR in RAW264.7 cells treated with or without hAMSCs-CM. Each experiment was repeated three times. Data represent as means ± SD. *p< 0.05; **p< 0.01, ***p< 0.001

Obesity is characterized as a chronic low-degrade inflammation. The adipokines secreted from adipose tissues were able to recruit a large number of macrophages to the adipose tissues and lead to inflammation. We further evaluated the roles of hAMSCs-CM in HFD-induced macrophage-mediated inflammation by examination of macrophages infiltration and inflammatory cytokines from epididymal adipose tissues in mice. Considering that macrophages polarization contributes to WAT inflammation, the inflammatory cytokines secreted from the M1 and M2 macrophages were detected by qPCR. The results showed that hAMSCs-CM remarkably reduced the mRNA levels of proinflammatory cytokines CD11C and TNFα, but enhanced that of anti-inflammatory cytokines ARG-1 and CD209A in WAT of the mice fed with HFD compared with the SFD group (Fig. 6A–D). As shown in Fig. 6E, hAMSCs-CM dramatically reduced HFD-induced F4/80 positive macrophages in WAT, in which the macrophages displayed classical crown-like structures. In addition, the p-STAT3/ARG-1 signaling pathway was also detected in WAT. The results showed that HFD significantly inhibited the protein expression of ARG-1 and increased the phosphorylation of STAT3 although the total protein levels of STAT3 were not altered. In comparison, hAMSCs-CM effectively reversed HFD-induced decreases of ARG-1 and increase of the phosphorylated STAT3 (Fig. 6F–H), suggesting that the anti-obesity of hAMSCs-CM might be involved in their anti-inflammation.

Furthermore, the effects of hAMSCs-CM on macrophages polarization were determined in RAW264.7 macrophages that were induced into M1 or M2 macrophage with LPS+IFNγ or IL4, respectively. The results showed that hAMSCs-CM inhibited the transcripts of M1 inflammation cytokines IL-6 and IL-1β and increased that of M2 anti-inflammation cytokines IL10 and ARG-1 in RAW264.7 cells (Fig. 6I–L). These results indicated that hAMSCs-CM might inhibit HFD-induced adipose tissue inflammation by activating STAT3/ARG-1 pathway or altering the macrophage polarization in HFD-induced obese mice.

## Discussion

Studies demonstrated that hAMSCs have a great potential for stem cell-based therapy in many diseases due to their pluripotency, low immunogenicity, no tumorigenicity, potent paracrine effects, and no ethical concern [23]. We previously observed that both hAMSCs and their CM efficiently repaired skin injury [24], inhibited hepatocellular carcinoma [25], and alleviated HFD-induced hyperglycemia in mice (unpublished data), suggesting that hAMSCs-CM plays a key role in the cells-based therapy by their paracrine effects through afftecting immunoregulation and anti-inflammation. It has been reported that exosomes from adipose MSCs ameliorated HFD-induced obesity in mice through anti-inflammation, improving insulin sensitivity and decreasing hepatic steatosis [27]. In the present study, we observed that hAMSCs-CM was able to improve HFD-induced metabolic disorders by reducing weight gain, improving insulin sensitivity, alleviating hepatic steatosis, and enhancing energy expenditure. In addition, our results also showed that hAMSCs-CM-mediated the inhibition of adipogenesis, enhancements of glucose metabolism and glycogen synthesis, and anti-inflammation contributed to their anti-obesity in HFD-induced obese mice. Furthermore, we demonstrated that the anti-obesity of hAMSCs-CM might be related to their multiple molecular mechanisms which were involved in activating AKT/GLUT4-mediated glucose metabolism, enhancing UCP1/PPARα-mediated energy expenditure, and increasing STAT3-ARG1-mediated M2 macrophage polarization during the development of obesity.

Obesity is characterized with hypertrophic adipocytes along with increased numbers of mature adipocytes which are differentiated from pre-adipocytes. PPARγ and C/EBPα are main regulators during early period of adipogenesis [32, 33]. It has been reported that downregulation of PPARγ and C/EBPα expressions ameliorated HFD-induced obesity through inhibiting adipogenesis [34]. Our results showed that hAMSCs-CM inhibited the differentiation of 3T3-L1 pre-adipocytes into mature adipocytes, and the mechanism might be related to hAMSCs-CM-induced downregulations of the lipogenesis genes such as PPARγ, C/EBPα, FAS, AP2, and SREBP1.

PPARα is highly expressed in liver, muscle, BAT, and heart and plays an important role in energy homeostasis [35]. PGC-1α usually forms a complex with PPARα to regulate many biological processes. Decreased PPARα and PGC-1α could downregulate the expressions of the genes related to the impairment of heart function and fatty acid oxidation. PPAR-α inhibitors can restore cardiac energy homeostasis and function [36]. Lipid accumulation in adipose tissues and low energy expenditure are characteristic features of obesity. Our study showed that hAMSCs-CM promoted energy expenditure by increasing VO_2_, VCO_2_, and water intake in HFD conditions, which was consistent with the upregulations of PPARα, PGC-1α in adipose tissues.

Lipid and glucose metabolism play a critical role in the energy homeostasis of adipose tissues [37]. GLUT4 is a key glucose transporter for regulating glucose intake in adipose tissues. Downregulation or disruption of GLUT4 was able to cause insulin tolerance, which further leads to severe diabetes and obesity [38]. AKT, a Serine/Threonine kinase which is activated by insulin growth cytokines, participates in lipid and glucose metabolism [30]. Our finding further demonstrated that hAMSCs-CM elevated lipid and glucose utilization by activating AKT phosphorylation. BAT contains abundant mitochondria and is acknowledged as thermogenesis organ. Studies indicated that PPARα activated UCP1 and promoted the expressions of the genes related to thermogenesis to repress HFD-induced obesity [39]. Study also showed that there were some “beige adipocytes” which were similar to brown adipocyte, in WAT, in which these special adipocytes were able to elevate the expressions of the thermogenesis genes such as UCP1, PPARα, PGC1α, Cidea, CPT1 and HSL to promote energy expenditure [40]. In the present study, we observed that hAMSCs-CM promoted UCP1 expression in BAT, indicating that the anti-obesity of hAMSCs-CM might be partially related to their promoting energy expenditure.

Obesity is a complicated metabolic syndrome in which the initiation and development of obesity may be associated with the disorders of the multiple organs/tissues in the body. Studies showed that human umbilical cord-derived MSCs exosomes improved insulin resistance and inflammation in type 2 diabetes rats [41], and the cytokines secreted from MSCs enhanced angiogenesis to protect kidney injury and wound healing through promoting angiogenesis [42, 43], suggesting that the cytokines or exosomes secreted from stem cells may play a key role in treating diseases. In the present study, we demonstrated that the anti-obesity of hAMSCs-CM was associated with their inhibiting lipid accumulation or adipogenesis, alleviating inflammation in adipose tissues, and promoting energy expenditure. However, due to the complexity of obesity, it is possible that multiple proteins or nuclear acid molecules in hAMSCs-CM may play the critical roles in their anti-obesity. Obviously, the key factors in hAMSCs-CM should be further identified in the future.

## Conclusion

Our study demonstrated that hAMSCs-CM significantly alleviated HFD-induced obesity in mice through suppressing lipid accumulation and adipogenesis in adipose tissues and liver, enhancing energy expenditure in multiple organs such as liver and muscle, and alleviating inflammation in adipose tissues. The underlying mechanisms of the anti-obesity of hAMSCs-CM might be involved in inhibiting PPARγ and C/EBPα-mediated lipid synthesis and adipogenesis, promoting GLUT4-mediated glucose metabolism, elevating UCP1/PPARα/PGC1α-mediated energy expenditure, and enhancing STAT3-ARG1-mediated M2-type macrophage polarization during the development of obesity. Obviously, our findings provided strong evidences that hAMSCs-CM has a great potential for the treatment of the metabolic disorders such as obesity clinically. Certainly, the key factors or components of the exosomes from hAMSCs-CM for their anti-obesity should be identified in the future.

## Supplementary Information

**Additional file 1: Table S1.** The primer sequences for qRT-PCR.

## Data Availability

The datasets used and/or analyzed during the current study are available from the corresponding author upon reasonable request.

## Abbreviations

hAMSCs: Human amniotic mesenchymal stem cells
hAMSCs-CM: Human amniotic mesenchymal stem cells-derived conditioned medium
hADSCs: Human amnion-derived stem cells
Sox2: SRY-box transcription factor 2
Oct-4: Octamer-binding transcription factor 4
SSEA-4: Stage-specific embryonic antigen 4
SFD: Serum-free DMEM
CHOW-D: Normal chow diet
ALT: Serum alanine transaminase
AST: Aspartate aminotransferase
TC: Total cholesterol
TG: Triglyceride
HDL-C: High-density lipoprotein cholesterol
LDL-C: Low-density lipoprotein cholesterol
TP: Total protein
CREA: Creatinine
BUN: Blood urine nitrogen
IF: Immunofluorescence
HE: Hematoxylin-eosin
PAS: Periodic acid-schiff
IBMX: 3-Isobutyl-1-methylxanthine
