# Supplementary material for: Human amniotic mesenchymal stem cells-conditioned medium protects mice from high-fat diet-induced obesity
Source: Stem Cell Res Ther. 2021 Jun 26;12:364. doi: 10.1186/s13287-021-02437-z (PMC8235646; doi:10.1186/s13287-021-02437-z)
Supplement: Supplementary file 1 — Additional file 1: Table S1. The primer sequences for qRT-PCR. [file 13287_2021_2437_MOESM1_ESM.docx]

| Arg-1 F | TTTTCCAGCAGACCAGCTTT |
| --- | --- |
| Arg-1 R | GGAACCCAGAGAGAGCATGA |
| IL-10 F | GCTCTTACTGACTGGCATGAG |
| IL-10 R | CGCAGCTCTAGGAGCATGTG |
| IL-6 R | TGGTACTCCAGAAGACCAGAGG |
| IL-6 R | AACGATGATGCACTTGCAGA |
| TNF-a R | GTGGAACTGGCAGAAGAGGCA |
| TNF-a R | AGAGGGAGGCCATTTGGGAAC |
| IL-1β R | AGGCTCCGATGAACAA |
| IL-1β R | AAGGCATTAGAAACAGTCC |
| CD209a F | GGGGCTCAACTTGTGGTCAT |
| CD209a R | GGTGTCATTCCAGCCGTCAT |
| CD206 F | GTCAGAACAGACTGCGTGGA |
| CD206 R | AGGGATCGCCTGTTTTCCAG |
| CD11c F | CTAGCACACGGTTCTCCCTG |
| CD11c R | GCTCCACTTTGGGTGGTGAA |
| PPARγ-F | GTGCCAGTTTCGATCCGTAGA |
| PPARγ-R | GGCCAGCATCGTGTAGATGA |
| CEBPα-F | TAGGTTTCTGGGCTTTGTGG |
| CEBPα-R | AGCCGTTAGTGAAGAGTCTCAGTTT |
| AP2-F | ACACCGAGATTTCCTTCAAACTG |
| AP2-R | CCATCTAGGGTTATGATGCTCTTCA |
| FASN-F | GGAGGTTGCTTGGAAGAG |
| FASN-R | CTGGATGTGATCGAATGCT |
| CD34-F | GGCCATTCAGCAAGACAACAC |
| CD34-R | GGAGCCGAATGTGTAAAGGACA |
| CD45-F | ATGGATCTCAGCAAACGGG |
| CD45-R | ATAACAGCTAACAGGAGGTTTGG |
| CD133-F | CCTTGTCCTTGGTAAGTGTTGT |
| CD133-R | CCTCTGGTGGGGTATTTCTTT |
| Nanog-F | AGAGTGTCGCAAAAAAGGA |
| Nanog-R | GTTCAGGATGTTGGAGAGTTC |
| OCT4-F | ATCCCTGAACCTAGTGGGGA |
| OCT4-R | CACTCGGACCACATCCTTCT |
| SOX2-F | AGTCTCCAAGCGACGAAAAA |
| SOX2-R | GGAAAGTTGGGATCGAACAA |
| CD29-F | CCGCGCGGAAAAGATGAAT |
| CD29-R | AAATGTCTGTGGCTCCCCTG |
| CD49-F | TGTGAGAAGGTTGACGAGA |
| CD49-R | GCAGAGCACCATCAGAGAG |
| CD105-F | TCCTCCCAAGGACACTTGTA |
| CD105-R | CGCCTCATTGCTGATCATAC |
| CD73-F | TGAAGTTGTGGGAATCGTTGG |
| CD73-R | TGTGCCATTGTTGCGTTCAT |

**Supplementary Materials**

Tan HR et al. Human amniotic mesenchymal stem cells-conditional medium protects mice from high fat diet-induced obesity

**Supplementary Table1. The primer sequences for qRT-PCR**
